# PIEZO1 is essential for the survival and proliferation of acute myeloid leukemia cells

**DOI:** 10.1002/cam4.6984

**Published:** 2024-02-09

**Authors:** Delphine Lebon, Louison Collet, Stefan Djordjevic, Cathy Gomila, Hakim Ouled‐Haddou, Jessica Platon, Yohann Demont, Jean‐Pierre Marolleau, Alexis Caulier, Loïc Garçon

**Affiliations:** ^1^ HEMATIM UR4666 Université Picardie Jules Verne Amiens France; ^2^ Hématologie Clinique et Thérapie Cellulaire, CHU Amiens‐Picardie Amiens France; ^3^ Service d'Hématologie Biologie, CHU Amiens‐Picardie Amiens France; ^4^ Division of Hematology/Oncology Boston Children's Hospital Boston Massachusetts USA; ^5^ Department of Medical and Population Genetics The Broad Institute of Harvard and MIT Cambridge Massachusetts USA

**Keywords:** acute myeloid leukemia, apoptosis, cell cycle, PIEZO1, proliferation, survival

## Abstract

**Introduction:**

Leukemogenesis is a complex process that interconnects tumoral cells with their microenvironment, but the effect of mechanosensing in acute myeloid leukemia (AML) blasts is poorly known. PIEZO1 perceives and transmits the constraints of the environment to human cells by acting as a non‐selective calcium channel, but very little is known about its role in leukemogenesis.

**Results:**

For the first time, we show that PIEZO1 is preferentially expressed in healthy hematopoietic stem and progenitor cells in human hematopoiesis, and globally overexpressed in AML cells. In AML subtypes, *PIEZO1* expression associates with favorable outcomes as better overall (OS) and disease‐free survival (DFS). If PIEZO1 is expressed and functional in THP1 leukemic myeloid cell line, its chemical activation doesn’t impact the proliferation, differentiation, nor survival of cells. However, the downregulation of *PIEZO1* expression dramatically reduces the proliferation and the survival of THP1 cells. We show that *PIEZO1* knock‐down blocks the cell cycle in G0/G1 phases of AML cells, impairs the DNA damage response pathways, and critically increases cell death by triggering extrinsic apoptosis pathways.

**Conclusions:**

Altogether, our results reveal a new role for PIEZO1 mechanosensing in the survival and proliferation of leukemic blasts, which could pave the way for new therapeutic strategies to target AML cells.

## 1 INTRODUCTION

Leukemogenesis in acute myeloid leukemia (AML) is a complex but poorly understood process, triggered by uncontrolled proliferation of immature cells. Numerous abnormalities, including molecular, disrupt the course of the cell cycle, the control of apoptosis and cell metabolism, eventually transforming hematopoietic cells into fast growing leukemic blasts.^1^, ^2^, ^3^, ^4^, ^5^ Considering the globally poor prognosis of AML, identification of new pathways involved in leukemogenesis that could be targeted in the future remains a priority for research teams.^6^

Mechanotransduction is an interesting avenue to explore in this area. For long identified as regulator of various physiological processes, many reports also highlighted its role during carcinogenesis.^7^ Indeed, cancer cells respond to mechanical stress by morphological changes, activation of signaling pathways, and changes in gene expression pattern that modify their properties of proliferation, migration, and response to treatment. However, so far, very few is known on the link between mechanotransdution and AML cells.^8^

In this report, we explored the potential role of PIEZO1 in AML pathophysiology. PIEZO1 is a widely expressed mechanotransducer capable of sensing the constraints of the environment has been involved in several physiological processes such as vascular development, regulation of blood pressure, regulation of red blood cell hydration status or immunity.^9^ Deregulation of PIEZO1 activity or expression has been described in many types of solid cancers, including hepatocarcinoma, gastric, colorectal, breast, and prostatic malignancies, mostly as an adverse prognostic factor.^10^, ^11^, ^12^, ^13^, ^14^ The putative mechanisms are multiple but often involve the deregulation of cell cycle and apoptosis.^10^, ^13^, ^15^ We and others identified PIEZO1 as expressed in leukemic cell lines such as K562 and UT7, where it regulates cell differentiation and proliferation through control of transduction pathways such as the ERK, STAT, and NFAT.^16^ Upon mechanical stimuli, PIEZO1 opens its central pore to passively allow the passage of cations, especially calcium (Ca^2+^), known to play a key role in the pathophysiology of AML blasts via several pathways, including calmodulin and calmodulin kinases (CAMKs). However, how calcium is precisely diverted by AML cells isn't fully elucidated yet.^17^, ^18^

Since (i) PIEZO1 senses mechanical forces and perception of the microenvironment is essential for AML cell survival and growth, (ii) its activation induces a calcium influx and calcium is essential for AML cell survival, we hypothesized the mechanosensor could play a role in the leukemogenesis of AML.^19^, ^20^, ^21^ For the first time, we describe the features of *PIEZO1* expression in healthy hematopoietic cells and human AML samples, and show the importance of mechanosensing in the proliferation and survival of AML cells.

## 2 MATERIALS AND METHODS

### 2.1 Cell culture

THP1, a cell line capable of differentiating into monocytes, was purchased from ATCC and grown in RPMI (Sigma Aldrich) supplemented with 10% decomplemented fetal calf serum (Eurobio) and 1% penicillin–streptomycin (Eurobio). THP1 monocytic differentiation was driven by exposing the cells for 72 h in the presence of 1 μM vitamin D (Sigma Aldrich) or for 24 h in the presence of 50 ng/mL phorbol‐12‐myristate‐13‐acetate (PMA, Sigma Aldrich) and then for 48 h with 25 ng/mL interleukin 4 (IL4, Miltenyi) and 25 ng/mL macrophage colony‐stimulating factor (M‐CSF, Miltenyi).

UT7‐EPO, erythroleukemia cell line, was cultured in minimal essential medium α (MEMα, Dominique Dutscher) supplemented with 10% FCS, 1% penicillin–streptomycin and 2 IU/mL erythropoietin (EPO, Roche).

Primary CD34^+^ cells were obtained from mononuclear cells derived from allogeneic apheresis and destined for destruction (cell therapy laboratory, CHU Amiens). CD34^+^ cells were purified using magnetic beads (Miltenyi Biotec Bergisch Gladbach, Germany) on the Miltenyi Biotec AutoMacs separator, according to the manufacturer's recommendations. The remaining CD34^−^ cell fraction was stained with lineage specific antibodies (cf list in Table S2) to sort mature hematopoietic subpopulations on the FACS Aria II sorter (Becton Dickinson) for assessment of *PIEZO1* expression. This negative fraction was sorted on cell surface markers and side scatter (SS). We isolated immature monocytic cells (monoblasts/promonocytes CD14^−^/CD64^+^), mature monocytes (monocytes CD64^−^/CD14^+^), lymphocytes (SS^Low^, CD45^High^), and immature granular cells (SS^High^, CD45^+^) (Figure S1).

### 2.2 Reagents

Yoda1 (Sigma Aldrich), chemical activator of PIEZO1, was used at a dose of 5 μM in cell culture, and at 20 μM for Ca^2+^ entry measurements; ethylene glycol‐bis (2‐aminoethylether)‐N,N,N′,N′‐tetraacetic acid (EGTA, Sigma Aldrich) at a dose of 4 mM, and Ionomycin (Sigma Aldrich) were used in culture.^16^ Quinoline‐Val‐Asp‐Difluorophenoxymethylketone (QVD, Sigma Aldrich) was used to inhibit pan caspases at a dose of 20 μM.

### 2.3 Lentiviral particle production and UT7/EPO cell transduction

A pool of four shRNA targeting PIEZO1 cloned into a lentiviral vector (PLKO3.1‐CMV‐tGFP, MISSION tool, Sigma Aldrich) were used as previously described.^16^ In experiments performed with separated shRNA#1 and #2, clone used was described in Table S1. Lentiviral particle production was performed in HEK293T cells as previously described.^16^ Lentiviral supernatant was ultracentrifugated for 1.5 h at 100,000 g at 4°C; THP1 cells were transduced using a multiplicity of infection (MOI) of 10 in the presence of 8 μg/mL polybrene (Sigma Aldrich). Cells were washed twice in 1X PBS (Eurobio scientific) 24 h after transduction. At D4 post‐infection, GFP cells were sorted on a FACS Aria II instrument (Becton Dickinson) and cultured for 3 additional days. Cell counts, flow cytometry, gene and proteins analysis were performed at D4, D7, and D9.

### 2.4 Flow cytometry

Multiparametric flow cytometry (MFC) was performed on MACSQuant flow cytometer (Miltenyi), and data were processed with FlowJo software (FlowJo v10, TreeStar Inc). THP1 monocytic differentiation was assessed at day 3. After washing in 1X PBS (200 μL per 10^5^ cells), cells (5 × 10^4^) were stained using panels of conjugated antibodies (Table S2), after exclusion of non‐viable cells with 7‐aminoactinomycin D (7‐AAD, Miltenyi Biotec) or DAPI (Tocris) positive. PIEZO1 labeling was performed on 2 × 10^5^ cells washed in 1X PBS and resuspended in PBS containing 2 mM ethylenediaminetetraacetic acid (EDTA, Santa Cruz) and 3% calf serum albumin (BSA, Sigma Aldrich), incubated for 1 h on ice. The cells were washed in the same buffer and incubated for 1 h on ice with the secondary antibody (Thermofisher). The cells were then washed and resuspended in 200 μL of PBS with 7‐AAD. Apoptosis was studied by combining labeling with 1:100 Annexin V APC (BD Pharmingen) and DAPI or 7AAD according to the manufacturer's recommendations and using Annexin‐specific buffer (BD Pharmingen). For cell cycle analysis, 2 × 10^5^ cells were washed and fixed according to the manufacturer recommendations (Thermofisher). After wash, cells were resuspended in 100 μL 1X Permwash (BD, Franklin Lakes NJ 07417) containing HOECHST (1/10000e) (Thermofisher) for 1 h in the dark at room temperature and then washed in 1X PBS.

### 2.5 Imaging flow cytometry

Flow imaging was performed on ImageStream®X Mark II (Amnis/Luminex) using INSPIRE™ software (200.1.388.0) to assess changes in THP1 calcium concentration. Cells (5 × 10^6^) were washed in 1X PBS and resuspended in RPMI alone with 4 μL of Fluo‐4 AM (Thermo Fisher). After 20 min incubation at 37°C, the cells were washed 3 times in PBS 1X without calcium/magnesium and then resuspended in PBS 1X with calcium and magnesium. Cells, after washing, were exposed either to 2 mM DMSO, 1 mM Ionomycin, or 10 and 20 μM Yoda1. Calcium flux measurements were performed with PBS 1X sheath fluid with and without calcium.

### 2.6 Quantitative reverse transcriptase‐polymerase chain reaction

Total RNA was extracted after treatment with DNAse I (Qiagen), using the Qiagen RNeasy Mini kit and following the manufacturer's recommendations. The amount of RNA extracted was measured using the Nanodrop ND 1000 spectrophotometer (Thermo Fisher Scientific). After reverse transcription to cDNA using the Thermofisher Reverse Transcription kit, gene expression levels were studied by RT‐qPCR using the Power SYBR Green assay (Thermo Fisher) on Quant Studio 7 (Applied Biosystems). The comparative C_T_ method was used for quantification of gene expression, and relative expression levels were calculated normalized to *GAPDH*. The primer sequences for the various genes studied are listed in Table S3.

### 2.7 Western blot

After extraction using RIPA lysis (Thermo Scientific) and extraction buffer (Sigma Aldrich) with 100X anti‐protease and anti‐phosphatase, the proteins were separated on a 15% Tris‐Glycine buffer Polyacrylamide gel (Thermo Fisher) and transferred to nitrocellulose membrane (Sigma Aldrich). The membranes were saturated for 1 h in 5% (w/v) TBS Tween 0.1% no‐fat milk. The membranes were incubated overnight at 4°C with the primary antibody solution in 5% (w/v) TBS Tween 0.1% no‐fat milk (detailed antibodies in Table S4). Membranes were then incubated with the secondary antibody solution in 5% (w/v) TBS Tween 0.1% no‐fat milk for 1 h at room temperature. After several washes with 0.1% TBS Tween, blots were visualized using chemiluminescent reagents (Super Signal Pico, Thermo Fisher) on the ChemiDoc Universal HoodII device (Bio‐rad).

### 2.8 Nanostring nCounter assay

The mRNA expression profile of THP1 cells after PIEZO1 KD was assessed with the Nanostring PanCancer pathway panel (NanoString Technologies), a multiplex analysis of more than 770 genes associated with cancer progression. The A260/A280 ratio of each sample RNAs was between 1,7 and 2,3 with a recommended input of 50 ng RNA minimum. Data were analyzed with the nSolverTM v4.0 software (NanoString Technologies).

### 2.9 Statistical analysis

Quantitative variables were compared by Student's *t‐*tests when biparametric, or Anova tests when multiparametric using the software GraphPad Prism 8 (GraphPad Prism Software Inc.). All numeric values were expressed as mean ± standard deviation and performed in triplicate at least. A two‐tailed *p*‐value <0.05 was considered significant. For survival analyses, Kaplan–Meier curves were generated and compared with the log‐rank test using the software R v4.3.2.

## 3 RESULTS

### 3.1 PIEZO1 is highly expressed in normal immature hematopoietic cells and blasts

**FIGURE 1:**
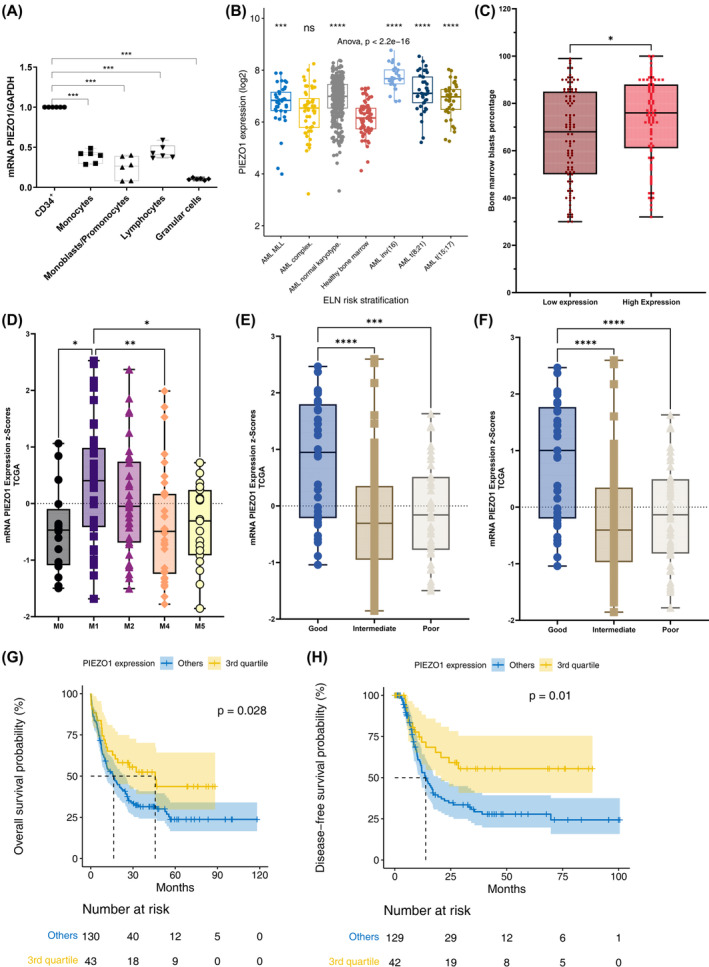
PIEZO1 expression pattern in normal hematopoietic cells and prognostic value in AML cohorts (MILE and TCGA). (A) *PIEZO1* mRNA expression was determined by quantitative reverse transcriptase‐polymerase chain reaction (RT‐qPCR) relative to *GAPDH* expression. Different cell fractions were sorted from peripheral hematopoietic stem cells and bone marrow as described in the methods section. *n* = 5; *** *p* < 0.001. (B) *PIEZO1* mRNA expression in 352 AML with normal karyotype and other abnormalities, 107 AML with favorable cytogenetic, 38 AML with 11q23 rearrangement, and 48 AML with complex karyotype compare to 73 healthy bone marrow (MILE GSE13159 cohort). (C) TCGA database (200 AML patients for whom genome or whole exome analysis was available) analysis of *PIEZO1* mRNA expression showed an increased bone marrow blast rate for patients *PIEZO1* expression over than median (*p* = 0.016). (D) TCGA database analysis of *PIEZO1* mRNA expression (Z‐score) according to cytological AML subtypes M1 (M3 subtypes were excluded) (*p* = 0.00057). (E) TCGA database analysis of *PIEZO1* mRNA expression (Z‐score) according to cytogenetics subgroups as defined by ELN 2017 classification (0.81 ± 1.09 vs. −0.21 ± 0.92 and −0.12 ± 0.86 for, respectively, intermediate and adverse cytogenetic, *p* = 5.3 × 10^−6^). (F) TCGA database analysis of *PIEZO1* mRNA expression (Z‐score) according to molecular profiles as defined by ELN 2017 classification (0.83 ± 1.07 vs. −0.23 ± 0.91 and −0.14 ± 0.88 for, respectively, intermediate and adverse molecular profiles, *p* = 1.5 × 10^−6^). (G) Overall survival of AML patients with high (higher than the third quartile) and low (below) *PIEZO1* mRNA expression (*p* = 0.028, from TCGA database). (H) Disease‐free survival of AML patients with high (higher than the third quartile) and low (below) *PIEZO1* mRNA expression (*p* = 0.01, from TCGA database). *p < 0.05; **p < 0.01; ***p < 0.001; ****p < 0.0001.

We first assessed *PIEZO1* expression across primary hematopoiesis, on purified cell fractions from peripheral blood and bone marrow (Figure S1). *PIEZO1* expression was significantly higher in immature CD34^+^ cells, a fraction that contains hematopoietic stem and progenitor cells (HSPCs), compared to differentiated cells of lympho‐granulo‐monocytic lineages, suggesting it plays a role early in the course of hematopoietic differentiation (Figure 1A).

We then analyzed available public databases to better characterize *PIEZO1* expression during normal and malignant hematopoiesis. The bloodspot website (https://servers.binf.ku.dk/bloodspot/) provides different databases including the MILE GSE13159 cohort, which records anonymized the transcriptome data from 73 normal bone marrow samples and 542 AML samples with normal cytogenetics and other abnormalities as core binding factor (CBF), acute promyelocytic leukemia (APL), mixed‐lineage leukemia (MLL) rearrangement, and complex karyotypes AML. *PIEZO1* was significantly overexpressed in AML bone marrow samples compared to healthy ones (Figure 1B). We then explored TCGA data for 200 AML patients with available whole genome or whole exome analysis. We found that *PIEZO1* expression dichotomized at median expression value was associated with some specific leukemia features such as a significantly higher marrow blast rate (Figure 1C), cytological immature AML subtypes (Figure 1D), favorable cytogenetics (Figure 1E), and favorable molecular profiles according to ELN 2017 classification (Figure 1F). Considering expression of *PIEZO1* by quartiles, AML patients with the highest expression of *PIEZO1* (above the third quartile, that is, the 25% of patients with the highest expression) had a significantly better overall survival (*p* = 0.028, Figure 1G) and leukemia‐free survival (*p* = 0.01, Figure 1H).

### 3.2 PIEZO1 is functional in THP1 myeloid cell line

Considering that expression of *PIEZO1* was high in normal immature hematopoietic cells and immature AML subtypes, we explored PIEZO1 function in AML blasts.

We first quantified *PIEZO1* expression in different AML cell lines, three with a myelomonocytic potential (THP1, HL60, and U937), and one committed to the erythroid lineage (K562). Using RT‐qPCR, we found that PIEZO1 was heterogeneously expressed in all of them (Figure S2A). *PIEZO1* expression was overexpressed in THP1 and K562, compared to normal CD34^+^ cells, while under expressed in HL60 and U937.

Thus, we selected THP1 as a relevant model to further explore PIEZO1 function in AML. Flow imaging using the Fluo4‐AM probe revealed that PIEZO1 mechano‐transductor was functional in THP1 cells, as its chemical activation induced a Ca^2+^ influx from the extracellular media (Figure S2B). Moreover, the survival of THP1 cells was directly dependent on extracellular Ca^2+^ availability, as shown by the drastic decrease in cell amplification (Figure S2C) and the increase in cell apoptosis observed in the presence of EGTA, an extracellular Ca^2+^ chelator (Figure S2D,E).

### 3.3 Chemical activation of PIEZO1 do not alter proliferation and differentiation of THP1

Chemical activation of PIEZO1 using Yoda1 had no impact on proliferation of THP1 cells (Figure S3A,B) or differentiation induced by vitamin D or PMA + IL4 + M‐CSF entitled “PMA” (Figure S3C,D). These results suggest an on/off effect, where calcium is essential for the survival of AML cells, whereas increasing the calcium influx doesn't affect their homeostasis.

### 3.4 PIEZO1 knock‐down significantly affects the survival of THP1

**FIGURE 2:**
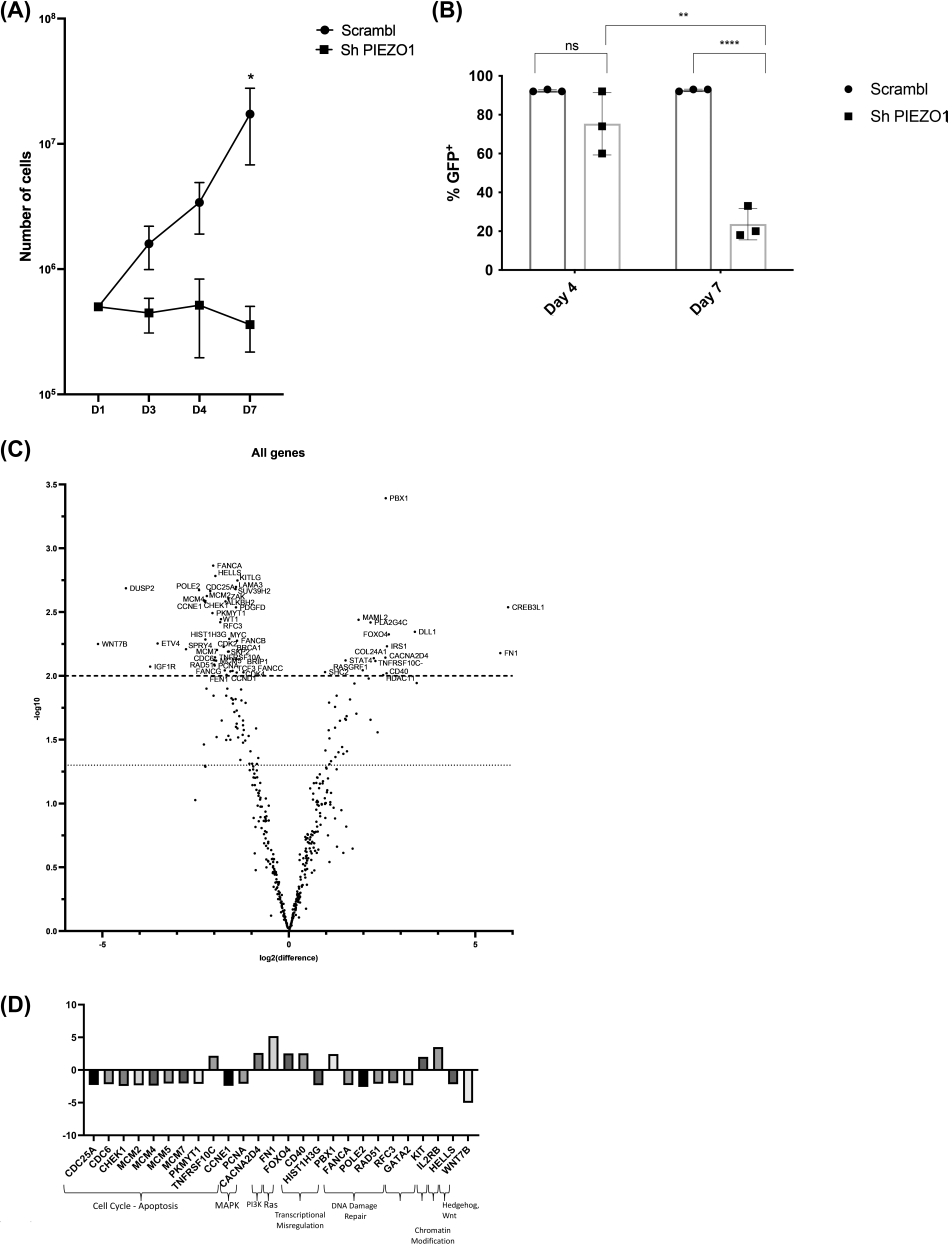
Effects of PIEZO1 KD on THP1 proliferation and transcriptomic data. All experiments were performed at less in triplicate; *** *p* < 0.001; ** *p* < 0.01; * *p* < 0.05. (A) THP1 total cell number at Day 7 in Scrambl shRNA (1,73 × 10^7^ ± 1,82 × 10^7^) and Sh PIEZO1 (3,61 × 10^5^ ± 2,48 × 10^5^). (B) Percentage of GFP^+^ cells at Day 4 (75.33% ± 16.04%) and Day 7 (23.57% ± 8.14%) in THP1 transduced with Sh PIEZO1 or with the Scrambl shRNA (92% ± 0.57 at Day 4 and XXXXX at Day 7). (C) Volcanoplot of differential gene expression between THP1 cells transduced with shPIEZO1 and Scrambl, panel of 770 genes from the panCancer pathway (NanoString Technologies). X‐axis is log2 (fold change), and Y‐axis is −log10 (adjusted *p*‐value). Significantly differentially expressed genes are indicated above the horizontal dashed line (adjusted *p*‐value = 0.01). (D) 26 genes were significantly deregulated (8 up‐ and 18 downregulated with a clear clustering between the two conditions) with an absolute value of Log2 (fold change) >2 and an adjusted *p*‐value <0.01.

To study the biological effects of PIEZO1 in THP1 cells, we knocked‐down PIEZO1 using a shRNA strategy. We observed a 53% ± 13 and 60% ± 4.79 decrease in PIEZO1 mRNA and protein level, respectively (Figure S4A,B). PIEZO1‐KD strongly inhibited the proliferation of THP1 cells, leading to a massive decrease in the percentage of GFP^+^ cells at D7 for the benefit of the non‐transduced GFP^−^ cells, whereas the GFP^+^ fraction remained stable in cells transduced with the shRNA control (Figure 2A,B).

To understand the mechanisms involved in the PIEZO1‐dependent survival of AML cells, we explored the transcriptional profile of THP1 cells after PIEZO1 KD, using the 770 genes nCounter panCancer pathways panel from NanoString Technologies (Figure S5). Most critical changes in gene expression corresponded to genes that regulate cell cycle and apoptosis, as well as DNA repair pathways (Figure 2C). By selecting genes with a fold change in expression greater than 2×log2 value after PIEZO1‐KD, we observed 26 dysregulated genes, 8 up‐ and 18 downregulated (Figure 2D).

### 3.5 Critical role of PIEZO1 in THP1 cell cycle and DNA damage response pathway

**FIGURE 3:**
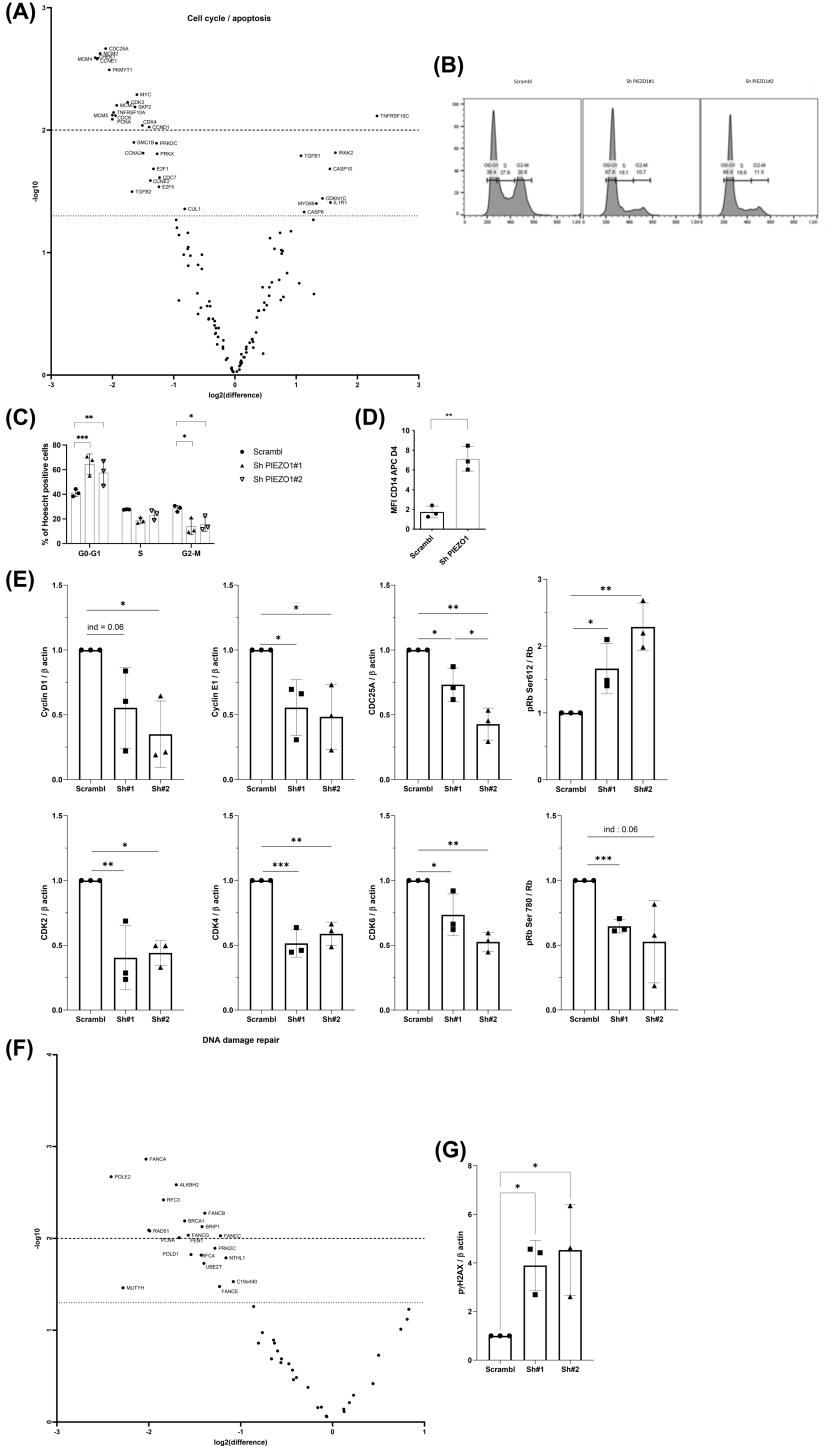
Effects of PIEZO1 KD on THP1 cell cycle and DNA damage pathway. All experiments were performed in triplicate; *** *p* < 0.001; ** *p* < 0.01; * *p* < 0.05. (A) Volcanoplot of expression of genes involved in cell cycle and apoptosis pathways deregulated after PIEZO1 KD, panel of 770 genes from the panCancer pathway (NanoString Technologies). X‐axis is log2 (fold change), and Y‐axis is −log10 (adjusted *p*‐value). (B) and (C) Cell cycle analysis of THP1 cells stained with Hoechst and analyzed by MFC revealed a blockage in G0/G1 phases (64.5% with Sh#1 and 57.4% with Sh#2 vs. 41% with Scrambl) and a significant decrease of G2/M cell cycle phases (13.8% with Sh#1 and 15.7% with Sh#2 vs. 28% with Scrambl) in PIEZO1 KD conditions. (D) MFC assessment of CD14 expression in THP1 cells 4 days after PIEZO1 KD (CD14 MFI Scrambl: 1.74 ± 0.6 and Sh PIEZO1: 7.12 ± 1.24). (E) Western blot analysis of key proteins of G0/G1 transition in THP1 cells, intensity normalized to beta‐actin and to Scrambl cells (Scr): cyclin D1 (Sh#1: 0.55 ± 0.31, Sh#2: 0.35 ± 0.26), cyclin E1 (Sh#1: 0.55 ± 0.21, Sh#2: 0.48 ± 0.25), CDC25A (Sh#1: 0.73 ± 0.13, Sh#2: 0.43 ± 0.12), CDK2 (Sh#1: 0.4 ± 0.25, Sh#2: 0.44 ± 0.1), CDK4 (Sh#1: 0.51 ± 0.11, Sh#2: 0.59 ± 0.09), and CDK6 (Sh#1: 0.74 ± 0.16, Sh#2: 0.53 ± 0.07) relative to β actin. Phospho Rb Ser‐780 (Sh#1: 0.65 ± 0.05, Sh#2: 0.53 ± 0.32) and phospho Rb Ser‐612 (Sh#1: 1.66 ± 0.38, Sh#2: 2.29 ± 0.36) were evaluated relative to Rb protein and to Scrambl cells. (F) Volcanoplot of expression of genes involved in DNA damage pathways deregulated after PIEZO1 KD. X‐axis is log2 (fold change), and Y‐axis is −log10 (adjusted *p*‐value). (G) Western blot of p‐γH2AX level in THP1 cells transduced with shPIEZO1 (3.895 ± 1.04 with Sh#1 and 4.53 ± 1.87 with Sh#2), normalized to beta‐actin and compared to the Scrambl.

We observed a global downregulation of genes involved in cell cycle (Figure 3A). Besides, gene expressions of DNA replication pathways were significantly decreased after PIEZO1‐KD, including several members of the origin complex which controls the replication fork, as MCM2, 4, 5, and 7, and CDC6. Altogether, transcriptional changes in cell cycle control and DNA replication pathways may explain the drop‐in proliferation of THP1 cells after PIEZO1 knock‐down. Cell cycle analysis using Hoechst staining by MFC revealed a significant blockage in G0/G1 phase of the cell cycle after PIEZO1 KD (Figures 3B and 2C). This blockage was associated with a significant increase in CD14 expression of THP1 cells, a marker of monocytic maturation, suggesting PIEZO1 might help maintain AML cells in an undifferentiated state (Figure 3D). To confirm that PIEZO1‐dependent control of cell cycle is essential for the survival of AML cells, we measured the expression of key proteins involved in critical checkpoints of cell cycle. Proteins involved in the G1/S transition were significantly downregulated, including cyclin D1, cyclin E1, CDK2/4/6, and CDC25A which was among the top downregulated targets in our transcriptomic study (Figure 3E). Downstream of CDC25A, we also observed a decrease in expression of cyclin D1, CDK2, CDK4, and CDK6 involved in the progression of the G1 phase of the cell cycle through the phosphorylation of the retinoblastoma protein (pRb, Figure 3E). We observed after PIEZO1 KD an increased phosphorylation of Rb at serine 612, known to occur as a response to DNA damage (Figure 3E). As our transcriptomic data showed a downregulation of genes associated with DNA damage response (Figure 3F), such as *RAD51* and *FANCA*, we wondered whether PIEZO1‐KD could induce a genotoxic stress and/or impair the cell response to DNA double strand breaks (DSB). DSB assessed by quantifying γH2AX phosphorylation were significantly increased after PIEZO1‐KD (Figure 3G).^22^ Of note, in response to DSB, γH2AX is phosphorylated by activation of the PI3K pathway, whose genes were also upregulated after PIEZO1‐KD. Gene expression of proteins involved in the repair of those DSB was deregulated, as *BRCA1* and *CHEK1* (Figure 2A), suggesting that PIEZO1 may regulate genomic integrity and response to DNA DSB.

### 3.6 PIEZO1 is involved in the negative control of the extrinsic pathway of apoptosis

**FIGURE 4:**
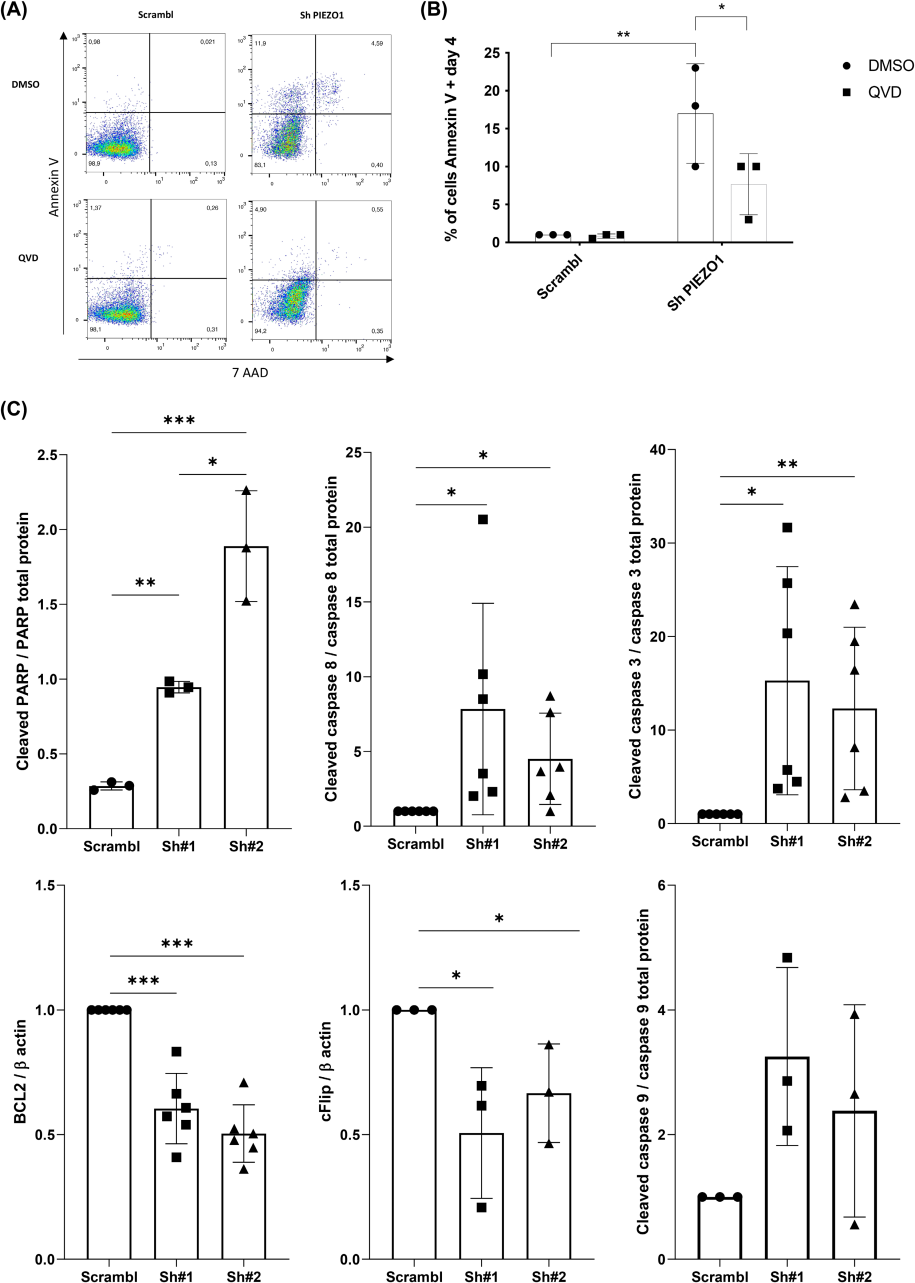
Effects of PIEZO1 KD on THP1 apoptosis. All experiments were performed in triplicate; *** *p* < 0.001; ** *p* < 0.01; * *p* < 0.05. (A) Assessment of Annexin V positive cells by MFC after PIEZO1 KD (17% ± 6.56 in shPIEZO1 vs. 1% in Scrambl cells). (B) Reversion assay of apoptosis adding 20 μM of the pan‐caspase inhibitor QVD in the medium at Day 4 post‐infection (7.67% ± 4.04 with shPIEZO1 vs. 1% with Scrambl). (C) Western blot analysis of proteins involved in apoptosis: PARP (Scrambl: 0.29 ± 0.03, Sh#1: 0.95 ± 0.04, SH#2: 1.89 ± 0.37), caspase 8 (Sh#1: 7.84 ± 7.1, Sh#2: 4.5 ± 3.1), caspase 3 (Sh#1: 15.3 ± 12.2, Sh#2: 12.3 ± 8.7), and caspase 9 (Sh#1: 3.25 ± 1.43, Sh#2: 2.38 ± 1.7) relative to their non‐cleaved forms, BCL‐2 (Sh#1: 0.6 ± 0.14, Sh#2: 0.5 ± 0.12) and c‐FLIP (Sh#1: 0.51 ± 0.26, Sh#2: 0.67 ± 0.2) relative to β actin.

Knock‐down of PIEZO1 triggered a massive apoptosis of THP1 cells, as shown by the significant increase in percentage of Annexin V positive cells (Figure 4A), which was partially relieved by the pan‐caspase inhibitor QVD (Figure 4B). A similar pro‐apoptotic phenotype was also observed after PIEZO1 KD in the erythroleukemic UT7/EPO cell line (Figure S6), showing that PIEZO1 role on leukemic cell survival was not restricted to a specific cell line. We then assessed by western blot the expression of major actors of apoptosis. We observed that apoptotic death mediated by PIEZO1 KD primarily involved the extrinsic pathway with cleavage of Caspase 3 and 8 while Caspase 9 remained non‐cleaved (Figure 4C). Besides, we showed a decrease in the expression of c‐flip (Figure 4C), which is known to negatively regulate Caspases 8 and 10 independently of the TNF pathway. Altogether, these results confirm that PIEZO1 integrity is involved to maintain cell survival by a negative regulation of the apoptosis extrinsic pathway.

## 4 DISCUSSION

We demonstrated here that PIEZO1 is mostly expressed in human immature healthy hematopoietic cells and is, in AML samples, correlated with a high percentage of bone marrow blasts. This is in line with our previous data from in vitro erythroid differentiation of CD34^+^ cells, where *PIEZO1* is highly expressed in early progenitors and then decreases along cell differentiation.^16^ In AML subtypes, a high expression of *PIEZO1* was associated with favorable outcomes, as well as favorable cytogenetic and molecular profile, although the underlying mechanisms aren't fully elucidated yet. However, despite this relative favorable profile, our results clearly highlight the role played by PIEZO1 in the survival and proliferation of AML cells through regulation of gene expression, including three main clusters corresponding to genes involved in the control of and genomic integrity, cell cycle, and apoptosis.

Many reports already highlighted the link between mechanical constraints at cell surface and gene expression, both in normal and cancer cells. The chromatin responds to a mechanical stress by promoting “mechanosensitive” gene expression.^23^, ^24^ Mechanosensors protect the nuclear envelop and DNA integrity from external stress.^25^ For example, in skin epidermis progenitor cells, PIEZO1 expression at cell membrane prevents stress‐induced DNA alterations.^26^ Interestingly, in HL60 AML cells, Blebbistatin, which targets the actomyosin contractibility, induced AML HL60 cells apoptosis, through the nuclear translocation of YAP and TAZ, two mechanotransducers that adapt gene expression to mechanical stimuli.^8^, ^27^ Thus, the DNA damages and the high apoptosis rate observed in THP1 cells after PIEZO1‐KD may be due to the loss of this protective mechanism that allows AML cells to adapt the gene expression profile to their environment. This led to a decreased expression of genes associated with DNA repair such as RAD51 and FANCA, as well as the high level of phosphorylation of ϒ‐H2AX, reflecting the DNA DSB and of Rb at Ser607, as a response to these breaks.^22^, ^28^

Cell response to genomic stress relies first on a cell cycle blockage.^29^ We demonstrate, in PIEZO1‐deficient THP1 cells, an accumulation of cells in G0‐G1 phase cell cycle. We observed a decrease in proteins controlling the cell cycle progression, including cyclin D and CDK proteins, most of which are regulated by calcium and ATP‐dependent phosphorylation. In particular, we observed at transcriptional level a drastic reduction of CDC25A, a phosphatase involved in the transition between G1 and S phases of cell cycle, by dephosphorylating CDK4 and CDK6 to favor entry into S phase.^30^, ^31^ Considering that the role for CDC25A in AML cell proliferation has been previously reported, inhibiting CDC25A activity could be an efficient strategy to target leukemic cells.

Considering the deficiency in DNA repair machinery, it is not surprising that THP1 cells blocked in G1 phase of cell cycle after PIEZO1‐KD underwent a massive caspase‐dependent apoptosis. DNA damages and the decreased Bcl2 expression are expected to activate the intrinsic apoptosis pathway, that is, cytochrome c release from mitochondria and cleavage of caspase 9. Surprisingly, we observed mainly here an activation of the extrinsic apoptosis pathway, as shown by the cleavage of caspase 8. This suggests that PIEZO1 is crucial to regulate apoptosis mediated by dead domain (DD) receptors pathway (such as TRAIL‐R, FAS, and TNF‐R). Since the FAS pathway is a well‐known regulator of T cell‐mediated apoptosis in AML cells along with the deregulation of TRAIL signaling, our data suggest that PIEZO1 protects AML cells against FAS/TRAIL dependent apoptosis, to promote the survival and proliferation of AML cells.^32^, ^33^ This may occur through PIEZO1‐dependent regulation of c‐Flip, a negative regulator of caspases 8 and 10 activation identified as a potential therapeutic target to sensibilize AML cells to apoptosis, and which was significantly downregulated in PIEZO1‐KD condition.^3^ Recently, a TRAIL receptor agonist showed a synergic effect in patient‐derived and AML cell lines when combined with the selective BCL‐2 inhibitor venetoclax.^34^ Together, these findings suggest PIEZO1 could be an interesting target to sensitize AML cells to apoptosis and eventually eradicate AML blasts.

To summarize, we report here for the first time a crucial role for PIEZO1 mechanosensor in the survival and proliferation of AML cells, by controlling cell cycle dynamics, response to DNA damages, and sensitivity to apoptosis in a calcium‐dependent way. If our data suggest that PIEZO1 could play a role in the emergence of AML and modulate the prognosis, the mechanisms linking PIEZO1 to the observed phenotype warrant further investigation.

## AUTHOR CONTRIBUTIONS

**Delphine Lebon:** Data curation (equal); formal analysis (equal); funding acquisition (equal); investigation (equal); methodology (equal); resources (equal); software (equal); supervision (equal); validation (equal); writing – original draft (equal); writing – review and editing (equal). **Louison Collet:** Data curation (equal); formal analysis (equal); funding acquisition (equal); investigation (equal); methodology (equal); software (equal); validation (equal); visualization (equal); writing – original draft (equal); writing – review and editing (equal). **Stefan Djordjevic:** Investigation (lead); writing – original draft (supporting); writing – review and editing (supporting). **Cathy Gomila:** Investigation (lead). **Jessica Platon:** Investigation (lead). **Hakim Ouled‐Haddou:** Conceptualization (supporting); investigation (supporting). **Yohann Demont:** Investigation (lead). **Jean‐Pierre Marolleau:** Investigation (supporting); methodology (supporting); supervision (supporting); validation (supporting); writing – original draft (supporting); writing – review and editing (supporting). **Alexis Caulier:** Investigation (supporting); methodology (supporting); writing – original draft (supporting); writing – review and editing (supporting). **Loïc Garçon:** Investigation (supporting); methodology (supporting); project administration (supporting); supervision (supporting); validation (supporting); writing – original draft (supporting); writing – review and editing (supporting).

## CONFLICT OF INTEREST STATEMENT

The authors declare no competing financial interests.

## Supporting information


Figure S1.

Table S1.
Table S2.
**Table S3**.
Table S4.

## Data Availability

Further information is available from the corresponding author upon request.
