# Supplementary figures and images for: PIEZO1 is essential for the survival and proliferation of acute myeloid leukemia cells
Source: Cancer Med. 2024 Feb 9;13(2):e6984. doi: 10.1002/cam4.6984 (PMC10854442; doi:10.1002/cam4.6984)

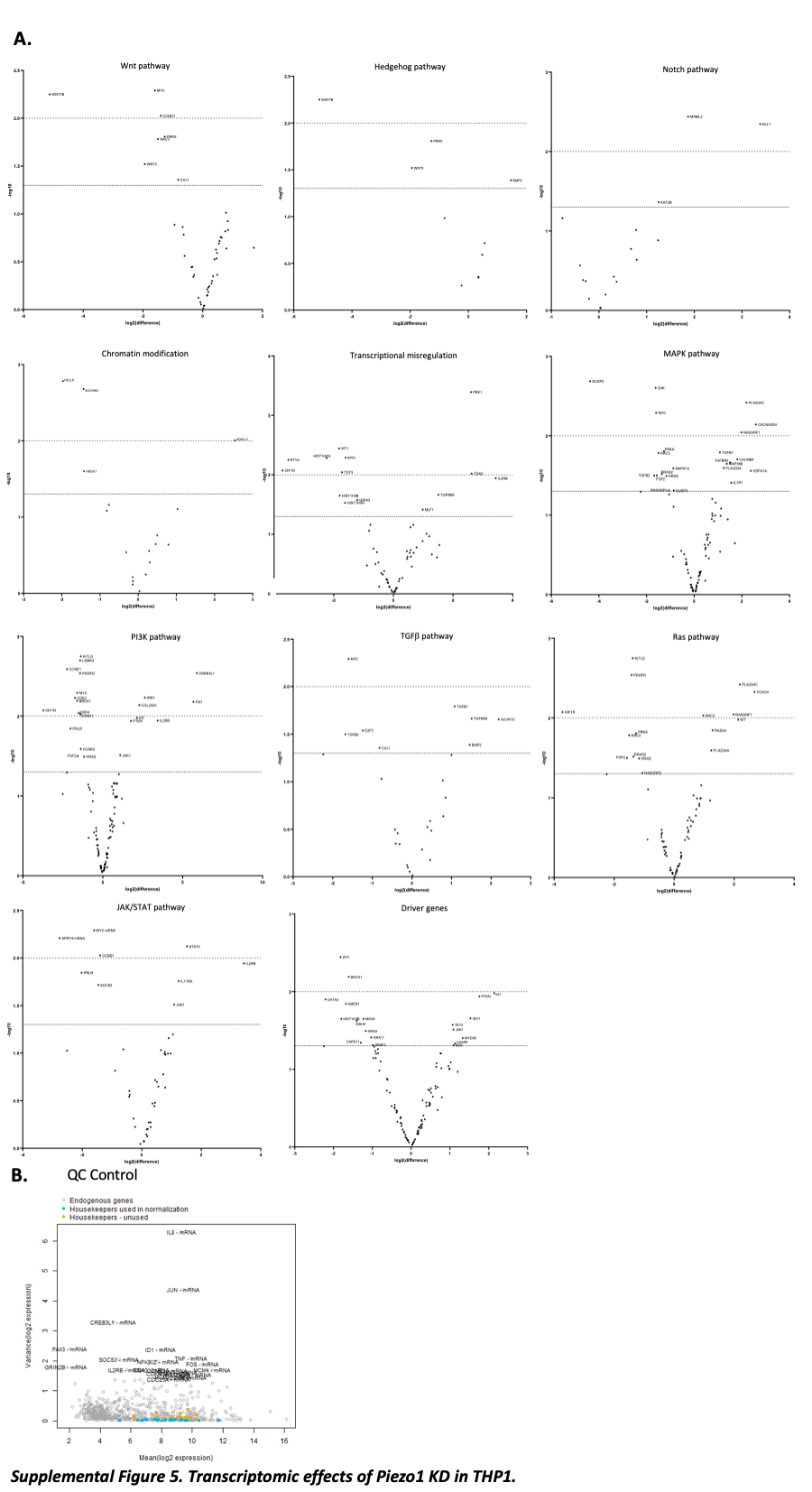

Supplement: Supplementary file 1 — Figure S1. [file CAM4-13-e6984-s002.zip › Diapositive10.tiff]

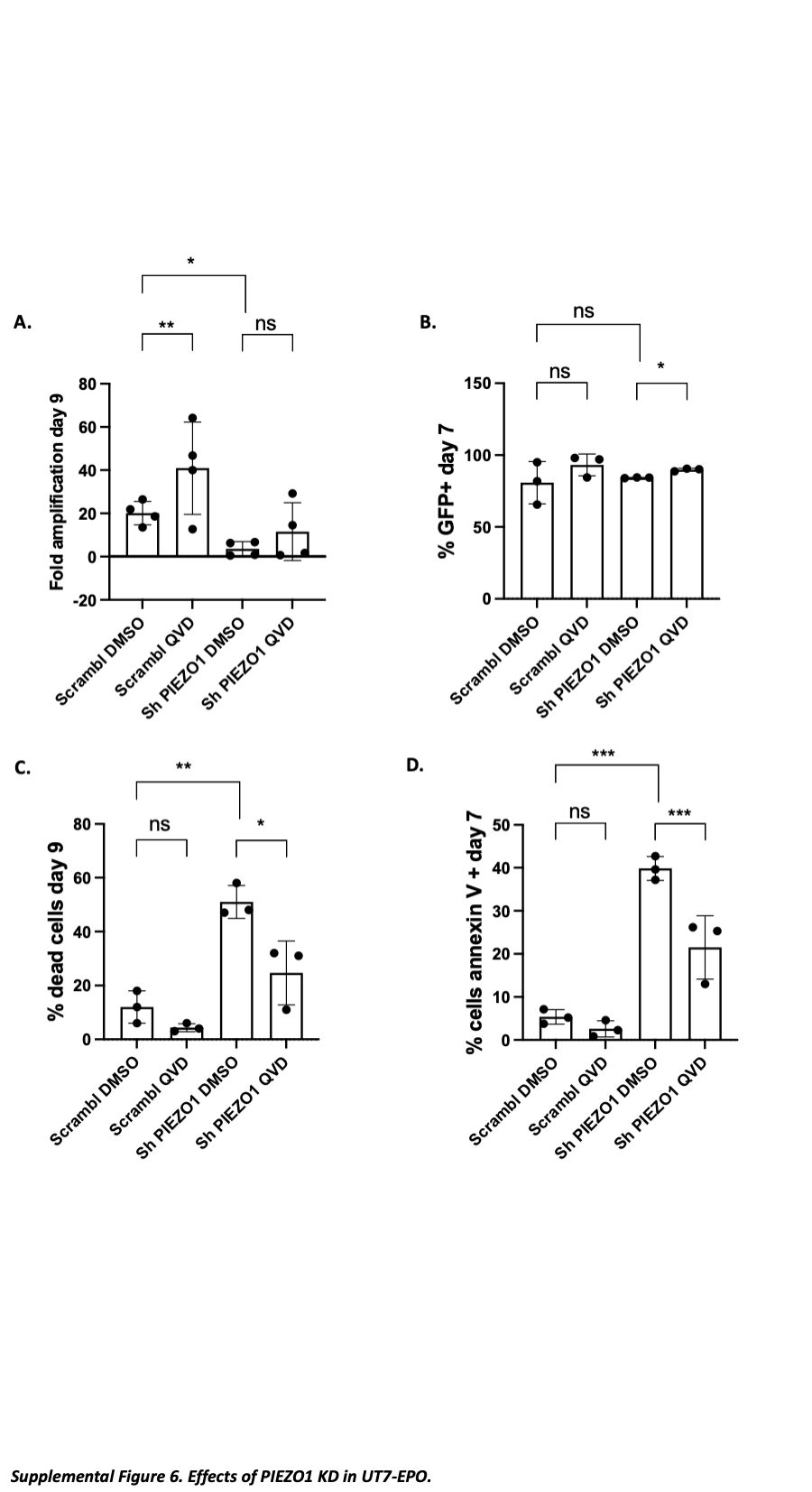

Supplement: Supplementary file 1 — Figure S1. [file CAM4-13-e6984-s002.zip › Diapositive11.tiff]

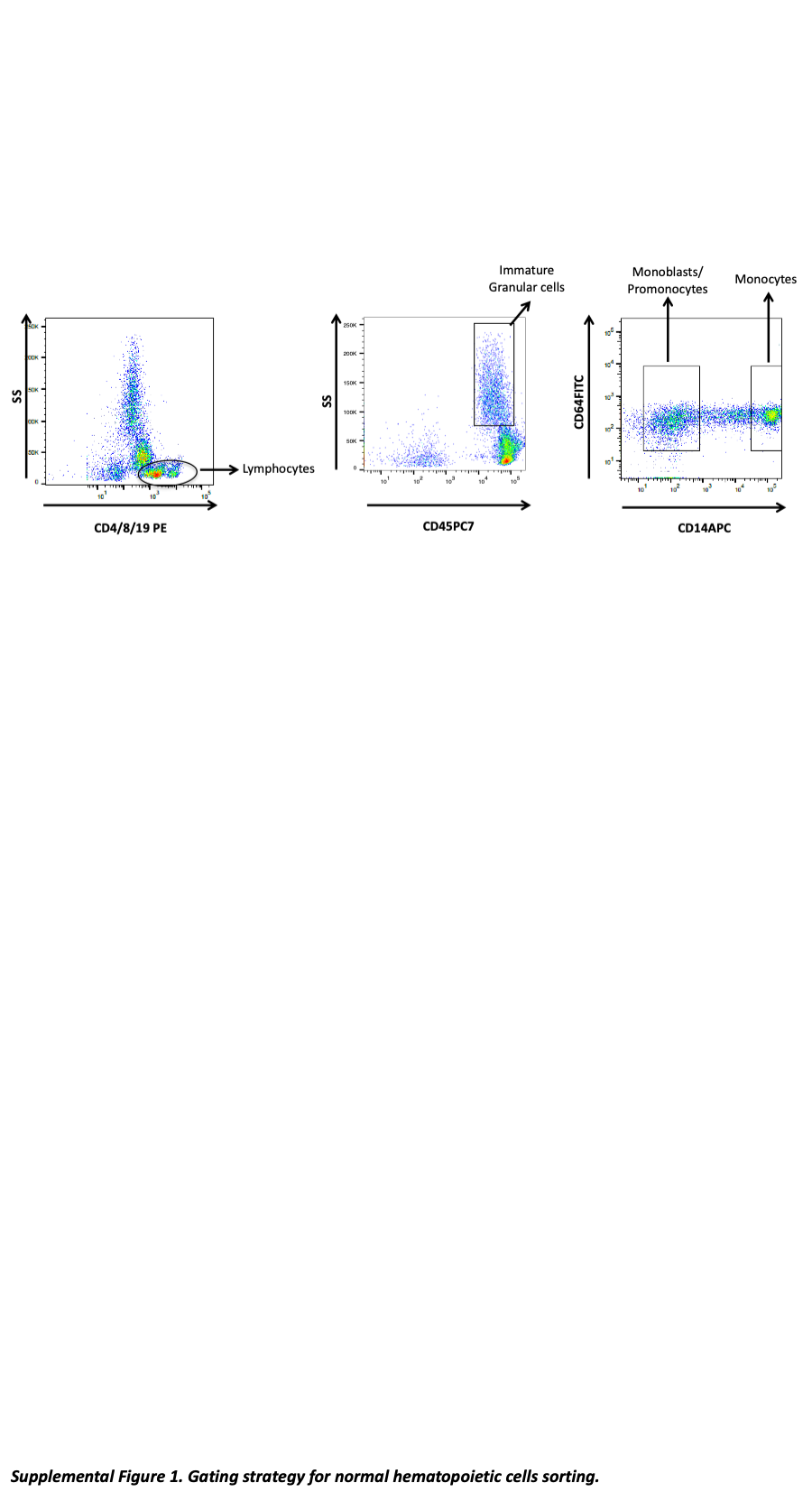

Supplement: Supplementary file 1 — Figure S1. [file CAM4-13-e6984-s002.zip › Diapositive6.tiff]

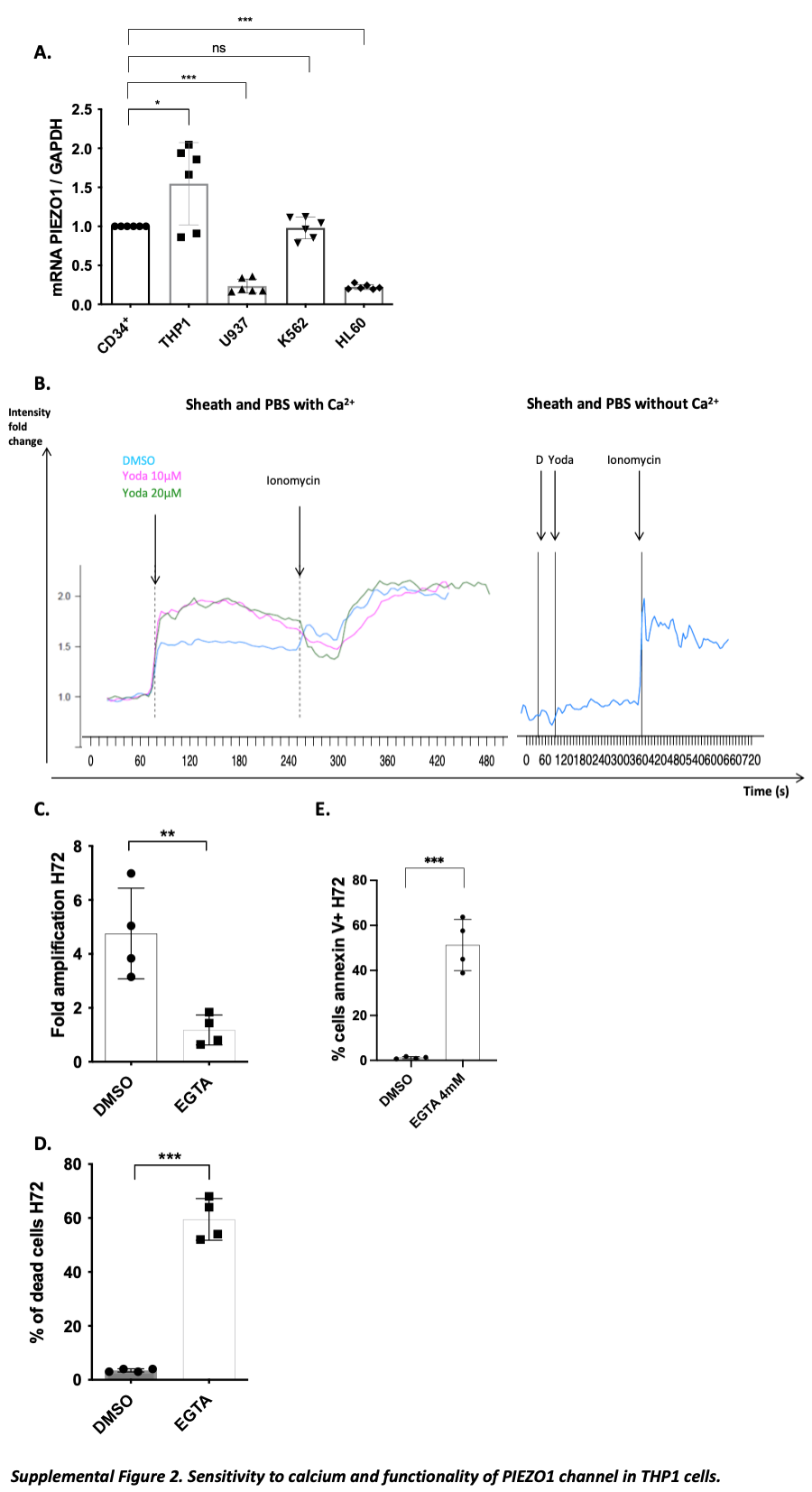

Supplement: Supplementary file 1 — Figure S1. [file CAM4-13-e6984-s002.zip › Diapositive7.tiff]

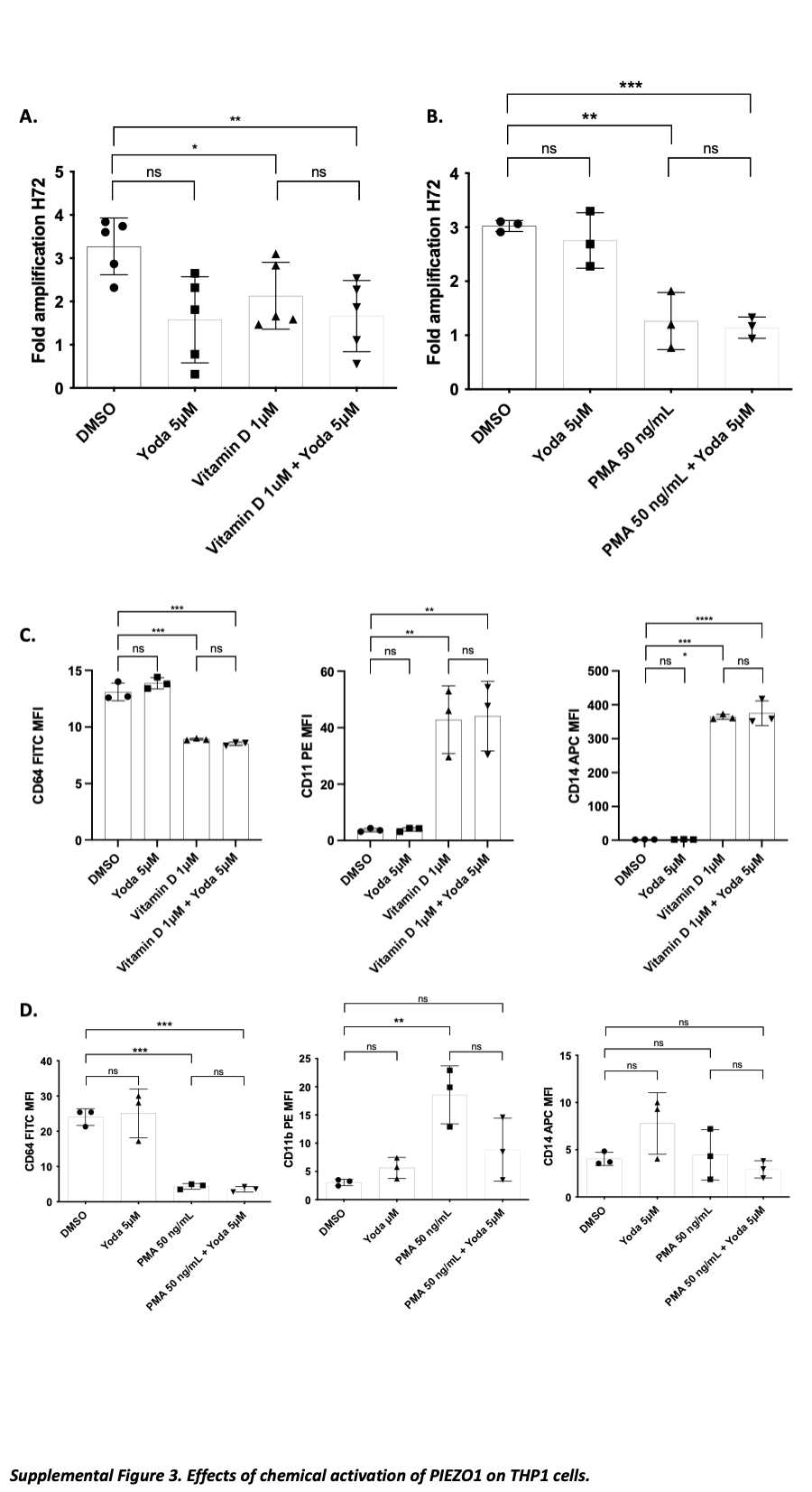

Supplement: Supplementary file 1 — Figure S1. [file CAM4-13-e6984-s002.zip › Diapositive8.tiff]

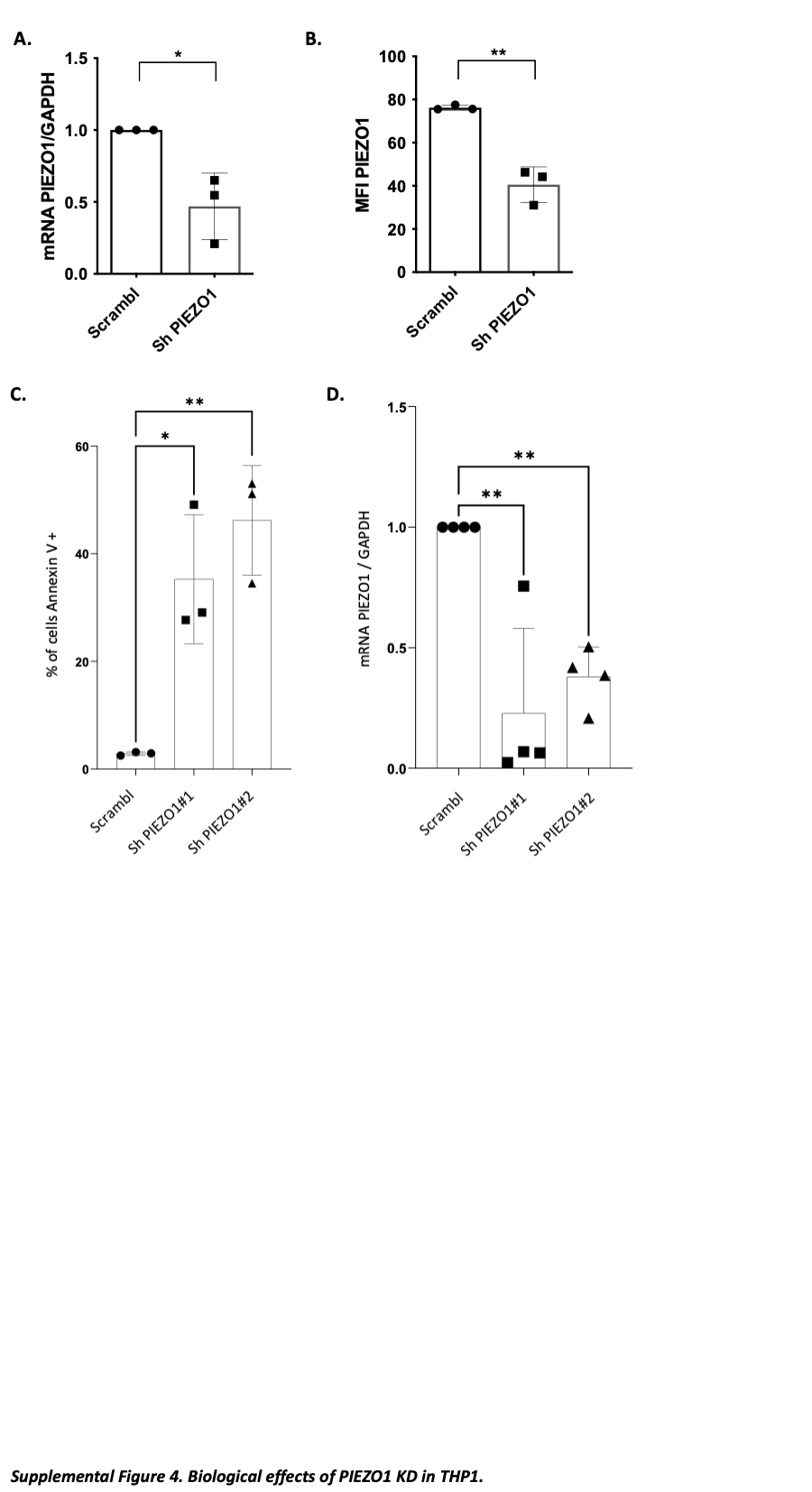

Supplement: Supplementary file 1 — Figure S1. [file CAM4-13-e6984-s002.zip › Diapositive9.tiff]
