# Supplementary material for: PIEZO1 is essential for the survival and proliferation of acute myeloid leukemia cells
Source: Cancer Med. 2024 Feb 9;13(2):e6984. doi: 10.1002/cam4.6984 (PMC10854442; doi:10.1002/cam4.6984)
Supplement: Supplementary file 2 — Table S1. Table S2. Table S3. Table S4. [file CAM4-13-e6984-s001.docx]

# Supplemental tables

Supplemental table 1. Location and targeted sequences (inserted in pLKO.1-CMV-tGFP vector) of the Sh-RNA anti PIEZO1.

| sh-PIEZO1 | Clone ID | Targeted sequence | Position |
| --- | --- | --- | --- |
| #1 | TRCN0000141308 | CCCTCTGCATTGATTATCCCT | Exon #22-#23 |
| #2 | TRCN0000142459 | GAAGACCACATCAGGTGGAA | Exon #39 |

Supplemental table 2. Characteristics and references of antibodies used for flow cytometry.

| Target molecule | Host specie | Clone | Dilution | Isotype | Conjugated | Source |
| --- | --- | --- | --- | --- | --- | --- |
| CD64 | Mouse | 22 | 1:40 | IgG1 | FITC | Beckman Coulter |
| CD4 | Mouse | 13B8.2 | 1:40 | IgG1 | PE | Beckman Coulter |
| CD8 | Mouse | B9.11 | 1:40 | IgG1 | PE | Beckman Coulter |
| CD19 | Mouse | J3-119 | 1:40 | IgG1 | PE | Beckman Coulter |
| CD14 | Human | REA 599 | 1:100 | IgG1 | APC | Miltenyi Biotec |
| CD11b | Mouse | Bear1 | 1:40 | IgG1 | PE | Beckman Coulter |
| CD45 | Mouse | J33 | 1:40 | IgG1 | PC7 | Beckman Coulter |
| CD33 | Mouse | D3HL60.251 | 1:40 | IgG1 | PC5 | Beckman Coulter |
| PIEZO1 | Rabbit | Polyclonal | 1:100 | IgG | Non conjugué | Proteintech 15939-1-AP |
| Secondary antibody | Rabbit |  | 1:200 | IgG | APC | Thermo Fisher |

Supplemental table 3. List of primers used for RT-qPCR.

| Genes | Orientation | Sequences 5’ 🡪 3’ |
| --- | --- | --- |
| PIEZO1 | forward | CATCTTGGTGGTCTCCTCTGTCT |
|  | reverse | GATGAGAGGGATGTGGATGCCAG |
| GAPDH | forward | TCGGAGTCAACGGATTTGGTCG |
|  | reverse | AGGGCATCCTGGGCTACACTGA |

Supplemental table 4. Characteristics and references of antibodies used for western blot.

| **Antibodies Targets** | **Antibodies species** | **References** |
| --- | --- | --- |
| Anti-human PIEZO1 | Rabbit | Abclonal (APC-087) |
| Anti-human Total/Cleaved Caspase8 | Rabbit | Cell signaling (9496S) |
| Anti-human Total Caspase3 | Rabbit | Cell signaling (9664S) |
| Anti-human Cleaved Caspase3 | Rabbit | Cell signaling (9662S) |
| Anti-human Total Caspase9 | Rabbit | Cell signaling (9502) |
| Anti-human Cleaved Caspase9 | Rabbit | Cell signaling (7237S) |
| Anti-human PARP | Rabbit | Cell signaling (9542S) |
| Anti-human BAX | Rabbit | Cell signaling (2772) |
| Anti-human BAK | Rabbit | Cell signaling (3814S) |
| Anti-human BID | Rabbit | Cell signaling (2002S) |
| Anti-human Bcl2 | Rabbit | Invitrogen (100/D5) |
| Anti-human p53 | Rabbit | Cell signaling (2527S) |
| Anti-human p-Histone γH2AX | Mouse | Millipore (JBW301) |
| Anti-human c-Flip | Rabbit | Cell signaling (3210) |
| Anti-human Cyclin E1 | Rabbit | Cell signaling (20808S) |
| Anti-human Cyclin D1 | Mouse | Santa Cruz Biotechnology (sc-593) |
| Anti-human CDK2 | Rabbit | Cell signaling (2546S) |
| Anti-human CDK4 | Rabbit | Cell signaling (12790S) |
| Anti-human CDK6 | Rabbit | Proteintech (14052-1-AP) |
| Anti-human CDC25a | Rabbit | Cell signaling (3652S) |
| Anti-human CDC25c | Rabbit | Cell signaling (4688S) |
| Anti-human Rb | Mouse | Cell signaling (9309S) |
| Anti-human Rb-Ser 780 | Rabbit | Cell signaling (3590S) |
| Anti-human Rb-Ser 612 | Mouse | Abnova (Mab0003) |
| Anti-human β-actin | Rabbit | Abcam (Ab8227) |
| Anti-human GAPDH | Mouse | Santacruz (32233) |

# Supplemental figures legends

Supplemental figure 1.Gating strategy for normal hematopoietic cells sorting.

Primary CD34^+^ cells were isolated, after magnetic sorting with CD34^+^ selection beads, from mononuclear cells derived from allogeneic apheresis. The remaining CD34^-^ cell fraction was stained with lineage specific antibodies in order to sort mature hematopoietic subpopulations (CD4^+^/8^+^/19^+^ population: lymphocytes; CD45^+^/SS^high^: immature granular cells; CD64^+^CD14^low^: monoblasts and promonocytes; CD64^+^/CD14^high^: monocytes) for PIEZO1 assessment.

Supplemental figure 2. Sensitivity to calcium and functionality of PIEZO1 channel in THP1 cells.

All experiments were performed at less in triplicate; *** p<0.001; ** p<0.01; * p<0.05.

(A) We assessed mRNA level PIEZO1 relative to GAPDH in different cell lines, three with a myelomonocytic potential (THP1, U937 and HL60), and a committed to the erythroid lineage (K562). Compared to the control CD34^+^ we found that PIEZO1 was heterogeneously expressed in all of them (THP1: 1.55±0.53; U937: 0.24±0.09; HL60: 0.22±0.03; K562: 0.98±0.14). n=6.

(B) Image flow cytometry using the Fluo4M probe revealed that PIEZO1 was functional in THP1. Cells (5 x 10^6^), after wash, were resuspended in RPMI alone with 2µM of Fluo-4 AM. Cells, after washing, were exposed to 2mM DMSO, or 1mM Ionomycin (used to raise intracellular Ca^2+^ level), or 10 and 20µM Yoda1, inducing after its activation a Ca^2+^ influx from the extracellular medium. Calcium flux measurements were performed with PBS 1X sheath fluid with or without calcium. We thus observed a Ca^2+^ influx from the extracellular medium with Yoda neither with DMSO. This influx is indeed from extracellular medium as, without Ca^2+^ in sheath and PBS, only Ionomycin caused a change in Ca^2+^ flow.

(C) When THP1 cells were cultured 3 days in EGTA contain medium (n=4), we could observe a decrease in proliferation with blue trypan staining (fold amplification respectively 4.75±1.68 for the control *vs*. 1.18±0.56 for EGTA condition).

(D) The rate of dead cells evaluated also with blue trypan staining was significantly higher in EGTA conditions (59.5%±7.72) than in control ones (3.5%±0.58).

(E) This cell death was mainly due to apoptosis as demonstrated by 7AAD and annexin co-staining (mean annexin V positive cells (%): respectively 0.3%±0.13 for the control *vs*. 44.98%±10.75 for EGTA condition).

Supplemental figure 3. Effects of chemical activation of PIEZO1 in THP1 cells.

All experiments were performed at less in triplicate; *** p<0.001; ** p<0.01; * p<0.05.

THP1 cell line was cultured in their usual medium and their monocytic differentiation was driven by exposing the cells for 72h in the presence of 1µM vitamin D or for 24 hours in the presence of 50 ng/mL phorbol-12-myristate-13-acetate and then for 48 hours with 25ng/mL IL4 and 25ng/mL M-CSF (called “PMA model”). Proliferation cell was measured by cell count in trypan blue staining. THP1 monocytic cell differentiation was assessed by flow cytometry with the loss of CD64 expression and acquisition of CD11 and CD14.

(A) Yoda1 used at a dose of 5µM did not impact THP1 proliferation. With vitamin D we observed a significant reduction of proliferation without significant impact of Yoda1.

(B) In the PMA model we also observed a significant reduction of proliferation with the differentiation, no more without significant impact of Yoda1.

(C) THP1 monocytic cell differentiation was observed with vitamin D with the loss of CD64 expression and acquisition of CD11 and CD14. Yoda1 had no impact on this differentiation.

(D) THP1 monocytic cell differentiation was observed with PMA model with the loss of CD64 expression and acquisition of CD11. Yoda1 had no impact on this differentiation.

Supplemental figure 4. Biological effects of PIEZO1 KD in THP1.

All experiments were performed at less in triplicate; *** p<0.001; ** p<0.01; * p<0.05.

(A) PIEZO1 KD significantly reduced mRNA of PIEZO1 relative to GAPDH (53%±13%).

(B) PIEZO1 KD significantly reduced the mean of fluorescence intensity of PIEZO1 protein (40.5±8.29 *vs.* 76.2±1.13).

(C) PIEZO1 KD induced a significant apoptosis of THP1 cells, compared to the control, not only by infection with the pool of ShRNA but also using separates ShRNA (mean of annexin V positive cells (%): 35.3%±11.97 with Sh#1 and 46.2%±10.18 with Sh#2 vs 2.85%±0.32 with Scrambl).

(D) RT-qPCR showed a significant reduction in PIEZO1 mRNA expression relative to GAPDH for Sh#1 (77.2%±35) and Sh#2 (62.1%±12).

Supplemental figure 5. Impact of PIEZO1 KD on THP1 cells: transcriptomic data.

KD PIEZO1 expression analysis on 770 genes using the pancancer pathway panel of Nanostring technology. All experiments were performed at less in triplicate.

(A) Volcanoplot showed the expression pattern of different pathways significantly affected by PIEZO1 KD.

(B) Volcanoplot of QC control with different housekeeping genes.

Supplemental figure 6. Effects of PIEZO1 KD in UT7-EPO cell line.

All experiments were performed at less in triplicate; *** p<0.001; ** p<0.01; * p<0.05.

UT7-EPO cells were transduced by the pool of 4 ShRNA using a MOI50. Cells were washed at day 4 post-infection, sorted according to the level of GFP expression and cultured for 3 additional days, with or without 20 μM of QVD.

(A) PIEZO1-KD significantly inhibited proliferation at day 9 (fold amplification: Scrambl+DMSO: 20.12±5.45; Scrambl+QVD: 40.93±21.39; Sh PIEZO1+DMSO: 3.59±3.37; Sh PIEZO1+QVD: 11.56±13.37).

(B) We observed no difference in the rate of GFP+ cells (Scrambl+DMSO: 80.83±14.77; Scrambl+QVD: 93.17±7.61; Sh PIEZO1+DMSO: 84.2±0.26; Sh PIEZO1+QVD: 89.8±0.96).

(C) PIEZO1 KD caused an increase of dead cells rate, partially reverted after QVD use, as assessed by trypan-blue (Scrambl+DMSO: 12±6; Scrambl+QVD: 4.33±1.53; Sh PIEZO1+DMSO: 51±6.08; Sh PIEZO1+QVD: 24.67±11.85).

(D) PIEZO1 KD induced a massive apoptosis of UT7-EPO, as shown by the increased percentage of Annexin V positive cells at day 7. QVD partially reverted this phenotype. Mean annexin V positive cells (%): Scrambl+DMSO: 5.37±1.71; Scrambl+QVD: 2.59±1.89; Sh PIEZO1+DMSO: 39.83±2.76; Sh PIEZO1+QVD: 21.5±7.38.
